# Angle-Adjustable Dynamic Hip Screw Plate for Unstable Trochanteric Fractures in Middle-Aged Patients: Mid-Term Outcomes and Return to Sport

**DOI:** 10.3390/jcm13040988

**Published:** 2024-02-08

**Authors:** Luca Andriollo, Giorgio Fravolini, Rudy Sangaletti, Loris Perticarini, Francesco Benazzo, Stefano Marco Paolo Rossi

**Affiliations:** 1 Robotic Prosthetic Surgery Unit—Sports Traumatology Unit, Fondazione Poliambulanza Istituto Ospedaliero, 25124 Brescia, Italy; luca.andriollo@poliambulanza.it (L.A.); giorgio.fravolini01@icatt.it (G.F.); rudy.sangaletti@poliambulanza.it (R.S.); loris.perticarini@poliambulanza.it (L.P.); francesco.benazzo@poliambulanza.it (F.B.); 2 Department of Orthopedics, Catholic University of the Sacred Heart, 00168 Rome, Italy; 3 Biomedical Sciences Area, IUSS University School for Advanced Studies, 27100 Pavia, Italy

**Keywords:** angle-adjustable dynamic hip screw, unstable trochanteric fracture, proximal femoral fracture, middle-aged, DHS

## Abstract

Background: There are conflicting results in the literature regarding the superiority of proximal femoral nails over dynamic hip screw (DHS) plates. The primary aim of this study is to evaluate mid-term post-injury patient-reported outcome measures (PROMs) and return to sport (RTS) in middle-aged patients treated with the DHS plate for unstable trochanteric fractures. Methods: Fifty-seven middle-aged patients (35–64 years) treated for proximal femoral fractures of type 31-A2 and 31-A3 according to the AO/OTA classification with the DMS Dynamic Martin Screw (KLS Martin Group, Jacksonville, FL, USA) between January 2017 and December 2019 were enrolled. Results: Forty-nine patients were included in this retrospective study, and the average age was 54.1 years (SD 8.4). The average follow-up duration at final follow-up was 60.5 months (SD 8.6). Post-operative complications included only one case of aseptic loosening of the implant, with a complication rate of 2%. There were no infections, peri-implant fractures, or other complications reported. Two out of the forty-nine patients (4.1%) required treatment with a total hip arthroplasty due to post-traumatic arthritis. The Harris Hip Score at final follow-up was 77.1 (SD 20.1), and the Western Ontario and McMaster Universities Osteoarthritis Index was 21.6 (SD 13.7). The overall rate of RTS was 57.7%. Conclusions: Treatment with DHS for unstable trochanteric fractures is a safe option in middle-aged patients, ensuring a good functional recovery.

## 1. Introduction

Hip fractures are associated with high mortality and morbidity and are among the most common injuries worldwide [1]. Of these, almost 50% are accounted for by trochanteric fractures of the femur, which can be classified as either stable or unstable [2].

Specifically, the Arbeitsgemeinschaft für Osteosynthesefragen/Orthopedic Trauma Association (AO/OTA) classification divides trochanteric fractures into three groups: 31A1 simple pertrochanteric fractures, with two fragments, considered stable after anatomical reduction; 31A2 multifragmentary pertrochanteric fractures with disruption of the medial cortex, considered unstable; and 31A3 intertrochanteric fractures with reverse obliquity. Subtrochanteric fractures, where the fracture line extends distally to the lesser trochanter, are classified as 32A [3].

Treatment includes intramedullary fixation with proximal femoral nails (PFNs) and extramedullary fixation with dynamic hip screw (DHS) plates with or without a trochanteric stabilization plate (TSP), fixed-angle blade plates, and proximal femoral locking plates [4]. 

The DHS plate was once considered the benchmark for treating trochanteric fractures, particularly those that are stable [5,6]. PFNs have become an increasingly popular fixation technique for these fractures since their introduction in the 1980s [7,8]. PFNs represent a more recent innovation, featuring funnel-shaped intramedullary nails that are slightly curved to match the shape of the proximal femoral diaphyseal trochanteric region. The primary benefit of PFNs lies in their ability to minimize surgical damage to both bone and soft tissue [9].

Nowadays, there are conflicting results in the literature regarding the superiority of PFNs over DHS plates [2,10,11]. In particular, in unstable trochanteric fractures with lateral wall damage, DHS plates in conjunction with the TSP play a critical role in providing a buttressing effect and preventing excessive fracture collapse, excessive medialization, limb shortening, and varus malalignment [4,12,13]. PFNs have been shown to have advantages such as improved patient return to pre-operative status, reduced intraoperative blood loss, and a lower incidence of complications [14,15]. In addition, PFNs can also be used in the treatment of unstable fractures and subtrochanteric fractures [16].

Hip fractures in young adults (<65 years) are generally the result of high-energy trauma, often associated with high-impact injuries such as car accidents, sports injuries, or falls. In contrast, the highest peak in the older population is due to low-energy secondary injuries [17]. 

The majority of the international literature focuses on the outcomes of trochanteric fractures in older patients, despite the fact that younger patients have a higher risk of mortality than older patients who suffer a hip fracture [1]. In addition, the existing literature focuses on the most common intracapsular hip fractures, with very few publications on outcomes following extracapsular hip fractures [18]. Moreover, considering young patients, there is a lack of data on returning to sports activities.

Aim of this study is to evaluate mid-term post-injury patient-reported outcome measures (PROMs) and return to sport (RTS) in middle-aged (<65 years) patients treated with the DHS plate for unstable trochanteric fractures. The secondary objective is to assess peri-operative and post-operative complications, including the rate of surgical reintervention.

## 2. Materials and Methods

Patients treated with an angle-adjustable DHS plate system between January 2017 and December 2019 were retrospectively evaluated. All procedures were performed at a single center by experienced trauma surgeons.

In this study, middle-aged patients who had been treated with the DMS Dynamic Martin Screw (KLS Martin Group, Jacksonville, FL, USA) for proximal femoral fractures of type 31-A2 and 31-A3 according to the AO/OTA classification were enrolled.

The inclusion criteria comprised individuals aged between 35 and 64 years (as per the definition of early and late middle-aged), a follow-up of at least 48 months (more than 4 years), and the availability of radiographic documentation (X-rays or CT scans) for both the trauma and post-operative follow-up. Exclusion criteria included pathological fractures, polytraumatized patients, open fractures, and loss of follow-up data.

Demographic and peri-operative data were collected, including the time from trauma to surgery, American Society of Anesthesiologists (ASA) classification, in-hospital complications, length of hospital stay, hemoglobin levels, blood transfusions, surgical duration, and the type of anesthesia administered.

The assessment also included the evaluation of acute complications, such as post-surgical local hematoma, vascular injury, or nerve injury, as well as follow-up complications, including readmission or reoperation rates and their respective causes, such as infection, screw cut-out, aseptic mobilization, non-union, and peri-implant fracture.

At the final follow-up, all patients underwent a clinical examination which included patient-reported outcome measures (PROMs) such as the Western Ontario and McMaster Universities Osteoarthritis Index (WOMAC) and Harris Hip Score (HHS). Additionally, RTS was assessed, both in terms of participation and performance, and associations were evaluated between peri-operative characteristics and RTS as well as documenting the types of sports that patients resumed at the final follow-up.

**Figure 1:**
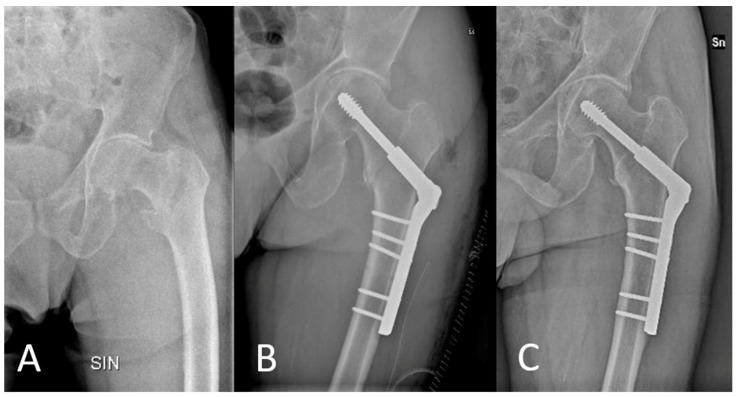
X-ray assessments at the time of trauma (**A**), immediately post-operatively (**B**), and at the final follow-up (**C**).

Radiographic assessment was also performed during the final follow-up to evaluate the presence of device mobilization or any progression of osteoarthritis (Figure 1).

Patients received routine venous thromboembolism prevention with low-molecular-weight heparin until full weight-bearing was resumed. Alternatively, chronic anticoagulant therapy was administered as an option. Furthermore, cefazolin was used as a routine peri-operative prophylactic antibiotic. In detail, cefazolin 2 g was administered intravenously 30 min before the surgical procedure and cefazolin 1 g intravenously every 12 h for 36 h following the surgery. The post-operative rehabilitation protocol was not consistent for all patients, with variations in weight-bearing and joint mobilization recommendations.

### 2.1. Surgical Technique

The patient is placed in the dorsal decubitus position on a radiolucent operating table. A 15 cm long straight, lateral skin incision is made two finger widths proximally to the tip of the trochanter major. To countersink the femoral cortex, a 4.5 mm drill bit is used. The aiming device, which can be adjusted between 135° and 150°, is used to position the guide wire. The guide wire is then inserted under image intensifier control, ensuring that it is centrally located in the femoral head’s mid-axis.

Once the guide wire is properly positioned, its length can be read off the scale of the measuring sleeve. After setting it to the measured value (−10 mm), the DMS combo reamer is drilled into the bone along the guide wire while being monitored by an image intensifier until the cone of the third stage has fully entered the lateral cortex. The tap is now screwed into a point 10 mm away from the cortex, optionally using the centering sleeve and the T-handle. The thread depth can be read directly from the mark on the centering sleeve. The length of the lag screw corresponds to the set drilling depth. To insert the lag screw, first attach it to the screwdriver and the connector before screwing it in with the safety inserter, 11 mm centering sleeve, and T-handle. After the lag screw has been properly positioned, the handle, safety inserter, and centering sleeve can be removed.

A plate of the proper length can now be passed over the screwdriver and onto the lag screw. Once the plate is in the proper position relative to the femoral axis, it is adjusted with the worm gear to correct any valgus or varus.

The worm gear is turned with a screwdriver until the plate is perfectly attached to the femur. The plate impactor is used to precisely adjust the DMS plate on the femur to ensure a secure seat. To secure the DMS plate to the femur, 4.5 mm cortical screws are used. A 6.5 mm cancellous screw can also be used for fixing the lesser trochanter in the plate hole directly underneath the worm gear. The fracture is finally compressed by inserting the DMS compression screw. Compression paths of up to 6 mm can occur in osteoporotic bone. The compression screw is removed after compression.

### 2.2. Statistical Analysis

Statistical analysis was performed using SPSS v18.0 (Chicago, IL, USA) by an independent statistician. Continuous variables were reported using averages and standard deviations (SD), while categorical variables were presented using frequency distributions and percentages. Biserial correlations were performed using a two-tailed test. The level of significance was set to *p* < 0.05.

## 3. Results

From January 2017 to December 2019, 57 patients were treated with the DMS Dynamic Martin Screw (KLS Martin Group, Jacksonville, FL, USA) for proximal femoral fractures of type 31-A2 and 31-A3 according to the AO/OTA classification. By the final follow-up, one patient (1.8%) had died from causes unrelated to the treatment, five patients (8.8%) met the exclusion criteria, and two patients (3.5%) were excluded due to a lack of data.

As a result, a total of 49 patients were included in this retrospective study; of these, 29 were male (59.2%) and 20 were female (40.8%). Nineteen cases (38.8%) involved the right hip and thirty (61.2%) involved the left hip. At the time of surgery, the average age was 54.1 years (SD 8.4). 

Table 1 contains specifics about the baseline demographics at the time of surgery.

Data on pre-traumatic health conditions are reported in Table 2.

The average time from trauma to surgery was 1.18 ± 0.7 days. Thirty-one (63.3%) patients received spinal anesthesia. Surgical procedures had an average duration of 110.8 ± 29.7 min. Cerclage wires were used in 18 patients (36.7%). Pre-operative hemoglobin levels averaged at 13.53 ± 1.29 g/L, while first-day post-operative hemoglobin was 9.83 ± 2.3 g/L and discharge hemoglobin was 9.74 ± 2.5 g/L. Packed red cells were transfused in 27 patients (55.1%).

On the first or second day, 16 patients (32.7%) were mobilized into a vertical position with partial weight-bearing. The average duration of hospital stay was 6.5 ± 3.1 days. One patient experienced a post-surgical hematoma, which was surgically drained, and another patient had prolonged diffuse paresthesia in the surgically treated limb. No further complications, such as deep vein thrombosis or pulmonary embolism, urinary tract infection, heart failure, pneumonia, acute kidney injury, and vascular injury, arose during hospitalization in the analyzed patient cohort.

Complete intraoperative data, including in-hospital complications, are reported in Table 3.

**Figure 2:**
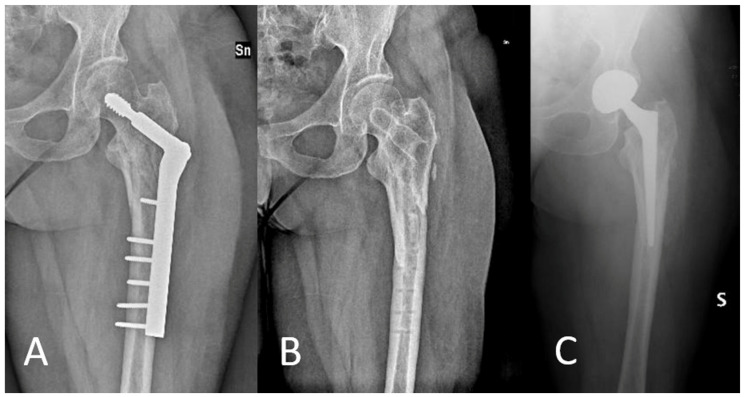
Patient underwent removal of synthesis devices and total hip replacement ((**A**): X-ray after treatment with DHS; (**B**): X-ray after removal of synthesis devices; (**C**): X-ray after total hip replacement surgery).

The final evaluation was conducted on 49 patients, taking into consideration factors such as death and exclusion criteria. The average follow-up duration at final follow-up was 60.5 months (SD 8.6). During follow-up, no complications such as local infection or peri-implant fracture were observed. Seven patients (14.3%) underwent the removal of the synthesis devices, including one case due to aseptic mobilization despite osseous healing. Two of the patients (aged 58 and 61 years, 4.1%) who underwent synthesis device removal were subsequently treated with total hip replacement for post-traumatic arthritis (Figure 2).

**Figure 3:**
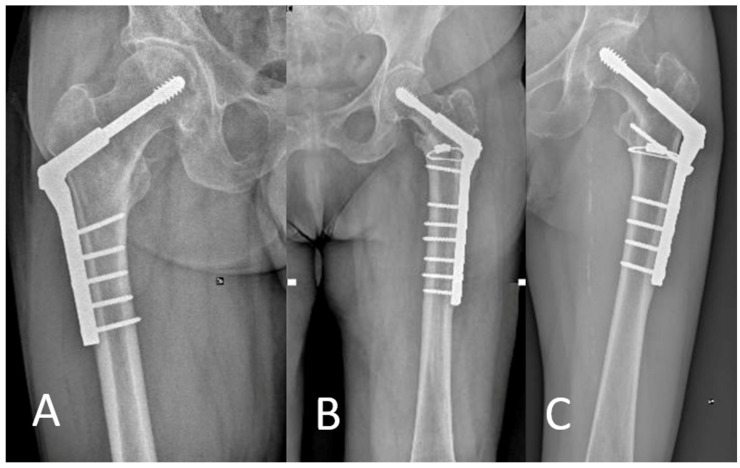
Three cases at follow-up: right hip with DHS (**A**) and left hip with DHS and cerclage (**B**,**C**); DHS = dynamic hip screw.

During radiographic assessment at the final follow-up, no further mobilizations or cut-outs were observed (Figure 3). In a single case, there was a presence of non-union of the greater trochanter, which remains clinically asymptomatic.

Of the patients enrolled in the study, 26 (53%) had been engaged in sports activities before the traumatic event. The overall rate of RTS was 57.7% (15 patients), defined as a return to participation, while the return to the same performance level occurred in 34.6% (nine patients). The average time for RTS for all sports was 34.3 weeks (SD 11.3).

The average HHS at final follow-up was 77.1 (SD 20.1). The average WOMAC score at final follow-up was 21.6 (SD 13.7). At the final follow-up, 15 (30.6%) showed excellent outcomes (HHS > 90), 14 (28.6%) good outcomes (HHS: 80–89), 13 (26.5%) fair outcomes (HHS: 70–79), and 7 (14.2%) poor outcomes (HHS < 70). Data related to PROMs and RTS are reported in Table 4.

The correlation between patient characteristics in the peri-operative period, PROMs at final follow-up, and correlation with RTS, intended as participation, was evaluated. As documented in Table 5, no statistically significant correlations emerged, except with PROMs at final follow-up.

Of the fifteen patients who resumed sports activities, seven (47%) practiced cycling, two (13%) football, four (27%) swimming, and two (13%) running. All the patients participated in sports at a non-competitive level.

## 4. Discussion

To the best of our knowledge, this is the first study that evaluates the use of the DHS plate in a cohort of middle-aged patients treated for unstable trochanteric fractures. Specifically, the type of DHS used is angle-adjustable, enhancing anatomical fit and respect for the femoral phenotype being treated.

The optimal treatment for unstable trochanteric and intertrochanteric fractures, whether to use a DHS plate or a PFN, remains a topic of debate in the literature. Specifically, unstable trochanteric fractures, especially intertrochanteric fractures, continue to pose a challenge to the orthopedic surgeon. These fractures are linked with increased rates of failure and the need for subsequent surgical revisions, regardless of the method of fixation employed. Moreover, the literature does not clarify the role of DHS treatment in middle-aged patients (<65 years old), who are underrepresented in studies that predominantly focus on an older population. This older group, however, has different types of issues and functional demands.

The cohort enrolled in this study exhibits characteristics that are distinct from those of other studies in the literature. Specifically, the average age is 54.1 (SD 8.4) years, and patients underwent surgery within the first 48 h following trauma, with an average time from arrival at the emergency department to surgery of 1.18 (SD 0.7) days. The discussion, which aims to assess the role of DHS in the treatment of unstable trochanteric fractures in middle-aged patients, is based on studies in the literature with the closest matching average age and demographic characteristics to the cohort evaluated in this study.

The surgical time was 110.8 (SD 29.7) minutes. In the literature, shorter operative times are reported. Sharma et al., in their study involving 29 patients with an average age of 62.3 years treated with DHS, reported an average time of 69.7 min [19]. Adeel et al. reported an average time of 58.7 (SD 7.8) minutes in 34 patients with an average age of 60.9 (SD 12.5) years [20]. Even shorter durations are reported in treatments using PFNs, with Xu et al., in a meta-analysis of 1889 patients, highlighting a statistically significant difference in the duration of surgery, with an average of 9.5 min less for treatments using PFNs [21]. Furthermore, if there is a need for an antirotation screw in addition to the DHS, the time increases further, as found by Mueller et al. in a study of 375 patients [22].

The DHS carries the drawback of requiring significant exposure and soft tissue stripping, which may lead to significant blood loss [23,24]. The patients evaluated in this study experienced an average decrease in hemoglobin of 3.8 g/L from their arrival at the emergency department to discharge, with 55% of the patients requiring at least one transfusion of packed red cells. In the literature, there is heterogeneity in this aspect, with some studies reporting a transfusion rate of up to 67.9%, while another study reported only 3.4% [9,19,21,25].

A meta-analysis published by Xu et al. reports data indicating less blood loss in patients treated with PFN [26]. A meta-analysis by Hao et al. compared different surgical techniques for intertrochanteric femoral fractures, focusing on intraoperative blood loss [27]. Their results highlighted the notable advantage of proximal femoral nail antirotation (PFNA), which had the lowest blood loss and the shortest operative time of the five treatments considered. After PFNA, the order of blood loss for each method was observed in ascending order: proximal cortical contouring plate, gamma nail, femoral head resection, and DHS. Strategically reducing blood loss during surgery not only minimizes the need for allogeneic blood transfusions, but also reduces the potential risks associated with transfusion reactions, disease transmission, and immunomodulation [28,29]. The reduced duration of surgery and lower incidence of blood loss may be attributed to the smaller incision and lesser muscle trauma. The PFN implant is placed using a minimally invasive technique that does not involve exposing the fracture site, in contrast to the DHS, which requires a larger incision [9,26].

Although not assessed in this study, several studies in the literature have evaluated the use of intraoperative fluoroscopy. DHS typically requires less fluoroscopic exposure compared to PFN [11,25,30,31]. Given that PFN procedures are performed through a minimally invasive approach, it can be expected that more fluoroscopic guidance would be necessary to ensure correct implant placement, achieve good stability, and reduce the risk of implant failure. For this reason, some authors suggest that DHS might be the preferable option for patients who must limit their exposure to radiation, such as younger individuals or those with multiple chronic conditions [26].

The cohort of patients presented in this study showed a shorter length of hospital stay compared to the data reported in the literature, with an average stay of 6.5 (SD 3.1) days. This early discharge was made possible due to meticulous pharmacological management of peri-operative pain, immediate rehabilitation, and a reduced rate of peri-operative complications. In fact, during hospitalization, only one patient developed a post-operative hematoma that required surgical drainage, and another patient experienced diffuse paresthesia in the operated lower limb. No other intraoperative complications were reported. The literature reports lengths of stay ranging from 10 to 14 days on average, with no statistically significant difference between patients treated with DHS and those treated with PFNs [19,22,25,32].

In the patient cohort featured in this study, at the final follow-up, post-operative complications only included one case of aseptic loosening of the implant, with a complication rate of 2%. There were no infections, peri-implant fractures, or other complications reported. The reoperation rate was 14.3%, with six out of seven patients undergoing removal of the fixation devices for reasons not associated with treatment failure.

Failures in cases treated with DHS alone have often been linked to excessive sliding of the compression screw, which can lead to the collapse or medial shift of the distal fracture fragment. This can occur even if the compression screw is ideally placed within the femoral head. Such failures are typically due to the loss of support from the calcar or to a deficiency in the lateral femoral wall, resulting in fracture collapse under load [33,34,35]. In the literature, non-union and implant failure are common complications associated with compromised fixation stability [36]. Sharma et al., in a study of twenty-nine patients treated with DHS, reported one case of loss of reduction and one case of implant failure, with a total complication rate of 7% [19]. Huang et al., in a study of thirty patients, reported two cases of fixation failure (6.7%) [9]. Yu et al., in a study of 110 patients, reported a non-union rate of 1.8% [35]. The incidence of post-operative complications such as fracture non-union, implant failure, revision of fixation failure, or arthroplasty did not show a significant difference between the use of PFNs and DHS. This finding is highlighted by the comprehensive meta-analysis published by Xu et al., conducted on 1889 patients, which reported a non-union rate of 1.7% in patients treated with PFNs and 2% in patients treated with DHS, and an implant failure rate of 2.5% with PFNs and 3.5% with DHS, with no statistically significant differences [26].

In the cohort in this study, two out of forty-nine patients (4.1%) required treatment with a total hip arthroplasty due to post-traumatic arthritis. Saudan et al. reported a prosthetic implantation rate at the final follow-up of 2.2%, while Parker et al. reported a rate of 4.3%, with both studies having a minimum follow-up of one year [25,30]. Acute arthroplasty treatment is a trending topic in the current literature, with some authors suggesting immediate replacement treatment, especially in cases of existing osteoarthritis or poor bone quality [37,38,39].

There are limited studies in the literature that have evaluated modern PROMs, such as HHS and WOMAC. In our study, the functional outcomes at follow-up were satisfactory, with an HHS of 77.1 (SD 20.1) and a WOMAC score of 21.6 (SD 13.7). Memon et al., in a study of 122 patients treated for unstable pertrochanteric fractures, reported an average HHS of 69.3 (SD 10) at 2 years’ follow-up [40]. In a study of 34 patients, Kassem et al. reported an HHS of 77.9 (SD 8.4) at 1 year follow-up [41]. The use of DHS in femoral neck fractures in adults aged between 18 and 69 years showed superior results, with an average HHS of 88 [42]. Watson et al. reported an average WOMAC score of 41 in a study involving 62 patients [43].

No study in the literature has evaluated RTS after proximal femur fractures. It can be hypothesized that the treatment we employed is less invasive on the gluteal muscles, which are necessary for sporting activity, when compared to PFNs. The findings of this study are thus capable of shedding light on the feasibility and success of resuming sporting activities after this type of surgery. This knowledge is crucial for both physicians and patients to understand the potential impact on an active lifestyle. However, the reported data are lower compared to the rates of RTS after elective hip arthroplasty in a middle-aged population [44].

This study has several limitations, including the lack of a control group, the limited number of patients assessed, the retrospective nature of the study, and the relatively brief duration of the follow-up period.

## 5. Conclusions

Treatment with angle-adjustable DHS plates for unstable trochanteric fractures is a safe option in middle-aged patients, ensuring good functional recovery comparable to outcomes achieved with intramedullary fixation or other extramedullary fixation systems.

Furthermore, this study represents the first publication on a cohort of patients treated with this device. Additional studies are required to assess complication rates and functional outcomes in comparison to patients treated with intramedullary nailing or with acute arthroplasty.

## Figures and Tables

**Table 1:** Baseline demographic data at time of surgery.

Patient Population	Number	%
Total no.	57	100
Died	1	1.8
Non-traceable	2	3.5
Exclusion criteria	5	8.8
Available	49	85.9
**Indication**	**Number**	**%**
31 A2	39	79.6
31 A3	10	20.4
**Sex**	**Number**	**%**
Male	29	59.2
Female	20	40.8
**Age**	**Average (Y)**	**SD**
	54.1	8.4
**Side**	**Number**	**%**
Left	30	61.2
Right	19	38.8

**Table 2:** Data on pre-traumatic health conditions.

ASA	Average	SD
	1.7	0.6
**ASA**	**Number**	**%**
1	17	34.7
2	28	57.1
3	4	
**BMI**	**Average (kg/m^2^)**	**SD**
	24.3	3.7
**Osteoporosis**	**Number**	**%**
	9	18.4

**Table 3:** Peri-operative and in-hospital data.

Time from Trauma to Surgery	Average (Day)	SD
	1.18	0.7
**Time from trauma to surgery**	**Number**	**%**
Day 0	8	16.3
Day 1	24	49
Day 2	17	34.7
**Surgical time**	**Average (min)**	**SD**
	110.8	29.7
**Cerclage**	**No. of patients**	**%**
	18	36.7
**Type of anesthesia**	**Number**	**%**
Spinal	31	63.3
General	17	34.7
**Hemoglobin (g/L)**	**Average**	**SD**
Pre-operative	13.53	1.29
Day 1	9.83	2.3
At discharge	9.74	2.5
**Transfusion of packed red cells**	**No. of patients**	**%**
	27	55.1
**Assisted verticalization with weight-bearing**	**Number**	**%**
1st day	5	10.2
2nd day	11	22.5
By discharge	10	20.4
No verticalization at discharge	23	46.9
**Days of hospitalization**	**Average (day)**	**SD**
	6.5	3.1
**In-hospital complications**	**Number**	**%**
	2	4.1

**Table 4:** Outcome data at final follow-up.

Final Follow-Up	Average (mos)	SD
	60.5	8.6
**Return to sport**	**Number**	**%**
Participation	15	53
Performance	9	34.6
**Return to sport**	**Average (w)**	**SD**
	34.3	11.3
**Clinical outcome**	**Average (pts)**	**SD**
HHS	77.1	20.1
WOMAC	21.6	13.7
**Quality score HHS**	**Number**	**%**
Excellent > 90	15	30.6
Good 80–89	14	28.6
Fair 70–79	13	26.5
Poor < 70	7	14.2

**Table 5:** Correlation between patient characteristics and return to sport.

	Return to Sport (N = 26)		
	Yes (N = 15)	No (N = 11)	*p* Value
Age	55.54(SD 9.47)	55.45 (SD 4.95)	0.805
BMI	23.65 (SD 3.12)	23.63 (SD 3.5)	0.644
ASA	1.76 (SD 0.6)	1.72 (SD 0.47)	0.787
Time from trauma to surgery	1 (SD 1.15)	1.63 (SD 0.67)	0.142
Surgical time	105.6 (SD 31.5)	119.6 (SD 24.25)	0.229
Days of hospitalization	5.61 (SD 1.8)	5.4 (SD 2.4)	0.917
Harris Hip Score	90.15 (SD 13.7)	67.54 (SD 13.23)	<0.05
WOMAC	13.76 (9.9)	27.1 (SD 10.4)	<0.05

## Data Availability

The data presented in this study are available on request from the corresponding author (privacy).
